# Optofluidic Lab-on-a-Chip Fluorescence Sensor Using Integrated Buried ARROW (bARROW) Waveguides

**DOI:** 10.3390/mi8080252

**Published:** 2017-08-17

**Authors:** Thomas Wall, Johnny McMurray, Gopikrishnan Meena, Vahid Ganjalizadeh, Holger Schmidt, Aaron R. Hawkins

**Affiliations:** 1 Electrical and Computer Engineering, Brigham Young University, Provo, UT 84602, USA; thomas.wall@byu.edu (T.W.); johnnymcmurray123@gmail.com (J.M.); 2 Baskin School of Engineering, University of California, Santa Cruz, Santa Cruz, CA 95064, USA; ggopikri@ucsc.edu (G.M.); vganjali@ucsc.edu (V.G.); hschmidt@soe.ucsc.edu (H.S.)

**Keywords:** optofluidics, lab-on-a-chip, fluorescence sensing, PECVD, SiO_2_, water absorption

## Abstract

Optofluidic, lab-on-a-chip fluorescence sensors were fabricated using buried anti-resonant reflecting optical waveguides (bARROWs). The bARROWs are impervious to the negative water absorption effects that typically occur in waveguides made using hygroscopic, plasma-enhanced chemical vapor deposition (PECVD) oxides. These sensors were used to detect fluorescent microbeads and had an average signal-to-noise ratio (SNR) that was 81.3% higher than that of single-oxide ARROW fluorescence sensors. While the single-oxide ARROW sensors were annealed at 300 °C to drive moisture out of the waveguides, the bARROW sensors required no annealing process to obtain a high SNR.

## 1. Introduction

Research in optofluidics, which is the combination of microfluidics with the fields of integrated optics and photonics, has recently seen the design and testing of many new and useful integrated micro-scale devices [1,2]. Many of these devices can be classified as lab-on-a-chip systems, that is, systems with the goal of miniaturizing macro-scale lab-like capabilities. For example, research is being conducted using optofluidic, lab-on-a-chip systems for on-chip cytometry [3], energy production [4,5], and genetic analysis systems [6].

This paper focuses on a new design for an anti-resonant reflecting optical waveguide (ARROW) fluorescence sensor, which is an optofluidic, lab-on-a-chip sensor using liquid-filled waveguides and capable of detecting the fluorescence of individual particles as they pass through a microscale excitation point. This type of sensor has already successfully detected the DNA from viruses and bacteria [7,8,9] as well as individual virus particles [10,11].

**Figure 1:**
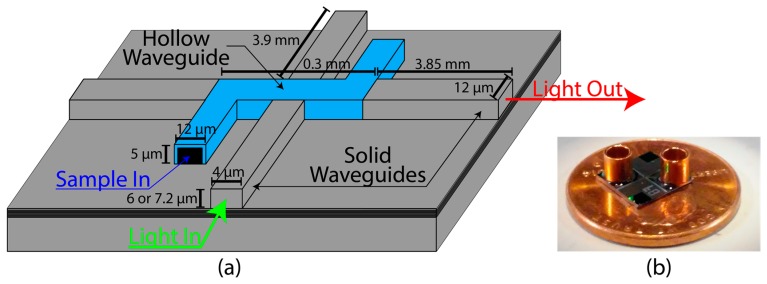
(**a**) Illustration of the basic anti-resonant reflection optical waveguide (ARROW) biosensor design. Figure is for illustrative purposes only and the features are not drawn in exact detail or to scale; (**b**) A completed ARROW biosensor resting on a penny (United States coin).

The basic design of the ARROW fluorescence sensor is shown below in Figure 1a. A hollow-core ARROW is intersected orthogonally by a solid-core ARROW, called the excitation ARROW. An aqueous solution, containing any fluorescent particles to be detected, flows through the hollow-core ARROW, where they emit an optical signal as they pass through the excitation point. In order to collect this signal off-chip, the hollow-core ARROW is integrated with another solid-core ARROW, called the collection ARROW, which allows the emitted light to be collected from the hollow-core ARROW and directed off-chip, where it is detected by an avalanche photodiode (APD). All of the waveguide dimensions of the fluorescence sensor were chosen in order to ensure efficient optical mode matching and coupling within the sensor. Figure 1b shows a completed ARROW fluorescence sensor resting on a standard penny (United States coin) for size reference. Copper beads were attached to the sensor to insert small samples into the hollow-core ARROW.

While the successful detection of various particles for several different applications has been demonstrated using this ARROW sensor design, all of the past ARROW sensors have shared a common problem: water absorption in the ARROW cladding layers has led to serious deterioration in waveguiding and subsequently poor fluorescence sensing results. This problem exists because the solid-core ARROWs are formed using SiO_2_ that was deposited via plasma-enhanced chemical vapor deposition (PECVD). It is well known that PECVD oxides are hygroscopic and will absorb moisture from their environment, causing key material changes in the oxides [12,13]. An example of one material change is that the index of refraction of the SiO_2_ increases by around 1.8% upon absorbing moisture [14]. The increase in the index of refraction is typically localized to a thin portion at the surface of the SiO_2_ film and creates an undesired, high index layer in the waveguide [15]. This high index layer readily forms after simply sitting out in standard atmospheric conditions for an extended period, and destroys the light confining characteristics of a rib waveguide [14]. This change is especially detrimental to the detection capabilities of the ARROW fluorescence sensor because it not only lowers the optical throughput of the waveguides, but it also introduces errant, unconfined excitation light that greatly increases the noise floor of the sensor.

**Figure 2:**
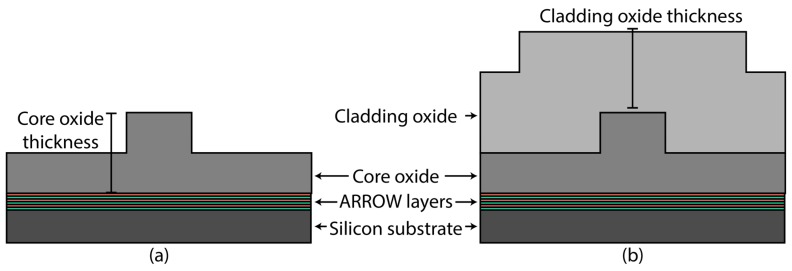
(**a**) Standard, solid-core, rib, single-oxide ARROW design; (**b**) Buried anti-resonant reflecting optical waveguide (bARROW) design with a double-oxide layer to protect the waveguiding core of the waveguide from water absorption.

Previously, an 8 h, 300 °C annealing process was used on each sensor to drive any moisture out of the ARROWs and achieve satisfactory sensing results; however, the moisture would only be expelled temporarily and over time the ARROWs would absorb moisture again and return to a poor waveguiding state [15]. While the waveguides could be annealed again to drive out the additional moisture, testing showed that each 300 °C annealing process caused irreversible damage to the waveguides, leading to a gradual decrease in optical performance. The annealing process is not a viable solution if the ARROW sensors are to be adopted for use outside of academic research. However, recent research has identified a new solution to mitigate the negative effects of water absorption in PECVD SiO_2_ waveguides by switching from a standard rib, single-oxide ARROW design to a buried ARROW (bARROW) design [16]. Figure 2 depicts (a) the single-oxide ARROW design and (b) the bARROW design with a double-oxide layer. The additional cladding layer over the top helps protect the guiding core from any water absorption effects. It should be noted that sensors were also fabricated that had a buried channel design, instead of a buried rib design. However, the deeper etch required to form the channel waveguide generally created a rougher surface than that created by the rib waveguide etch. The additional roughness made the growth of a cladding layer over the top more difficult, leading to lower yields and high losses from additional scattering points.

In this study, bARROW fluorescence sensors were fabricated, along with single-oxide sensors, and used to detect fluorescent microbeads. As expected, the measured SNR of an unannealed, single-oxide sensor was very low, due to water absorption, and increased dramatically once the moisture was expelled from the sensor’s oxide with a 300 °C anneal. However, the sensors using the bARROWs yielded higher average SNRs without the need for any post-fabrication anneals. It should be noted, that integrating bARROWs into the sensors initially introduced major cracking in the oxide layers and in the structure of the hollow channels on the sensors. A new PECVD process recipe was developed to fabricate sensors without any cracking. Design and testing of the sensor chips was complemented with FIMMPROP (Photon Design, Oxford, UK) simulations to ensure good optical coupling into the liquid-core section under realistic growth conditions.

## 2. Materials and Methods

**Figure 3:**
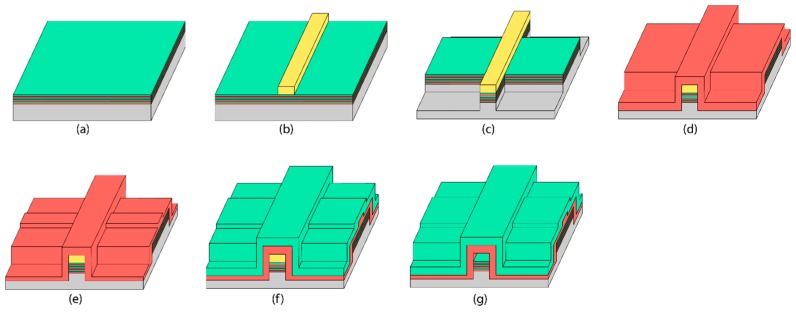
Illustrations of the basic fabrication steps to make an ARROW biosensor: (**a**) ARROW layers; (**b**) sacrificial sore; (**c**) pedestal etch; (**d**) solid core oxide; (**e**) rib etch; (**f**) cladding oxide; (**g**) sacrificial etch. These figures only include key elements of the sensor to illustrate fabrication processing and do not represent its entire geometry.

This section describes the basic fabrication process for the bARROW fluorescence sensor. There are seven major steps involved in the process that are illustrated below in Figure 3. First, the six bottom ARROW layers were deposited onto a silicon substrate. These layers were alternating SiO_2_ and Ta_2_O_5_ thin films of 265 nm and 102 nm, respectively, that were sputtered onto the substrate. Their thicknesses were designed to provide a highly reflective optical buffer layer between the waveguides and the optically absorptive silicon substrate [17,18,19]. Next, 12-µm wide sacrificial cores were patterned using 5-µm thick SU8 photoresist. These sacrificial cores are used as a temporary structural layer that will later be etched out to create a hollow channel. Next, a self-aligned pedestal etch step (SAP), described in further detail in References [20,21], was used in order to raise the waveguides up on a pedestal above the substrate. The presence of this pedestal helps enhance the structural integrity of the hollow ARROW waveguides and improve the yield in fabrication. This step is shown in Figure 3c.

A PECVD oxide layer was then deposited directly over the top of the sacrificial cores. This oxide makes up the structure of the hollow ARROW waveguides, but also serves as the material used for the solid-core ARROWs. This layer is referred to as the core oxide and was deposited with an index of 1.51 and a height of either 6 µm or 7.2 µm. All of the PECVD oxide layers were deposited in the same PECVD chamber (PlasmaLab 80 Plus, Oxford Insturments, Abingdon, UK), which is a dual power supply design, including both a low frequency (LF) and high frequency (HF) power supply. These power levels can be adjusted on each of these power supplies, allowing for greater control in tuning the index and stress of the films produced.

The next step, illustrated in Figure 3e, was an etch to define the rib waveguide geometry of the solid-core ARROWs. The rib feature was patterned with a nickel mask using standard photolithography processing and etched with an RIE/ICP dry etch tool (Phantom III, Trion Technology, Tempe, AZ, USA) to form a rib that was half the height of the core oxide thickness. The excitation solid-core ARROWs were patterned to be 4 µm wide in order to keep the excitation spot size small, but still ensure decent optical coupling with the optical fiber. The collection solid-core ARROWs were 12 µm wide to ensure good mode matching and coupling with the hollow-core ARROW. The last step before etching out the hollow-core ARROWs was to deposit a low index cladding over the ARROWs to bury and protect them from water absorption effects. There are two main requirements for the cladding layer to be a successful protective layer against any negative water absorption effects. First, the cladding layer must have a lower index of refraction than the core oxide for total internal reflection (TIR) waveguiding to occur between the two oxide layers. This requires that the cladding layer have an index of refraction at least 1.8% lower than the index of refraction of the core oxide, which ensures that even upon water absorption in the cladding layer, its index of refraction will remain lower than that of the core oxide. The bARROWs in this study had a core index of 1.51 and a cladding index of 1.448. The second condition is that the cladding layer should be thick enough to prevent any water absorption reaching the core oxide of the bARROW or close enough to couple evanescent light from the core into the cladding layer. The exact thickness needed depends on the mode FWHM, the density, and stress of the oxide used. In this study, all the cladding layers were 6 µm thick, which proved sufficiently thick to prevent negative water absorption effects from occurring in the sensors.

**Figure 4:**
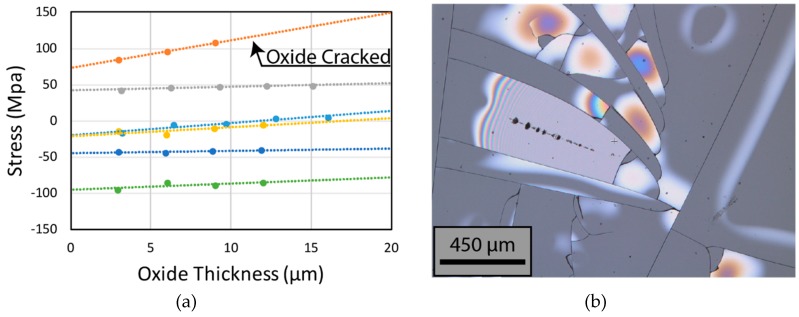
(**a**) Graph showing a relationship between intrinsic film stress and film thickness for six different plasma-enhanced chemical vapor deposition (PECVD) process recipes; (**b**) Microscope image of cracking in the highest stress oxide recipe (shown in orange) around 9–10 µm of growth.

The nature of PECVD oxide deposition is that individual PECVD chambers can behave rather differently than others. In our specific chamber, typically lower index process recipes grow with a higher intrinsic stress value. The first attempts to deposit low index cladding layers over the sensors led to significant cracking in the devices, especially when the hollow channels were etched out. This was due to the fact that the additional cladding layer effectively doubled the thickness of the oxide layers on the sensor. A study of stress versus film thickness was conducted on our PECVD chamber for several different process recipes. The stress in the films was measured by determining wafer bow using an optical 3D profilometer (Zeta 20). The results are shown in Figure 4a, and they revealed that the tensile stress in the films tended to increase with thickness for each recipe.

In fact, cracking occurred in the higher stress, low index recipe after 12 µm of growth. The cracking is shown in Figure 4b, and occurred on a flat substrate with no features or hollow channels with weak structural points. The situation is not the same for the sensors, which possess a more complex topology and have weaker hollow channels running through them. Cracking occurs even with lower stress values in these structures. A new process recipe was developed, which possessed a very low intrinsic stress value (~−12 MPa) at 6 µm of thickness, and still had a low index of refraction (1.448). bARROW sensors were successfully fabricated without any cracking using this new low stress, low index process recipe. All of the PECVD process recipes used are shown below in Table 1. The last step in the fabrication process was to expose the ends of the sacrificial cores and etch them out using an active piranha acid, which is a mixture of 3:2 H_2_O_2_:H_2_SO_4_.

**Figure 5:**
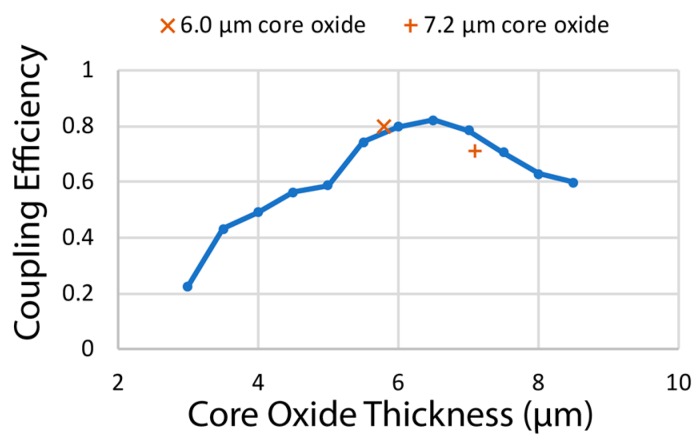
Simulation of the coupling efficiency in buried anti-resonant reflecting optical waveguide (bARROW) fluorescence sensors based on core oxide height. The blue line represents simulations using exact design dimensions, while the “×” and “+” represents simulations conducted after measuring the actual dimensions of completed bARROW sensors.

The addition of the thick oxide cladding layer to form bARROWs represented a major design change in the integrated waveguides of the fluorescence sensors and required detailed optical study to determine suitable dimension designs. One important measure of the sensors is their optical coupling efficiency, or the power percentage of excitation light that successfully enters the hollow-core bARROW from the excitation ARROW. The coupling efficiency was simulated versus core oxide thickness to determine appropriate core oxide thicknesses for bARROW fluorescence sensors. The simulations were run using the finite difference method on FIMMPROP software (Photon Design, Oxford, UK). Figure 5 shows the results of the simulation, with the blue line indicating the coupling efficiency calculated using ideal design parameters for feature sizes and only varying the core oxide thickness. The geometry simulated was altered to match expected, real-life features in the waveguides, including crevices that form during PECVD growth [21].

**Figure 6:**
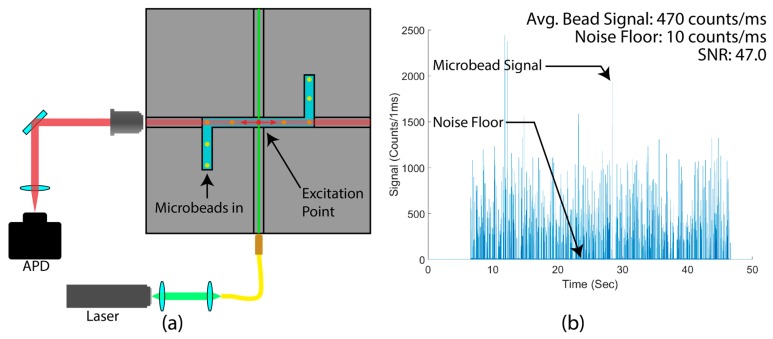
(**a**) Experimental setup for testing the signal-to-noise ratio (SNR) of the various ARROW biosensors; (**b**) Sample data from a buried anti-resonant reflecting optical waveguide (bARROW) fluorescence sensor with a core oxide thickness of 6.0 µm.

After bARROW sensors were fabricated, their feature sizes were measured using an SEM and simulations were run using the exact dimensions measured. These results are indicated on Figure 5 by the “×” and the “+” sign. The results of the simulations show that the coupling efficiency of the bARROW fluorescence sensors is quite forgiving with respect to core thicknesses, and that it is expected that sensors with a core oxide thickness between 5.5 µm and 7.5 µm will possess high coupling efficiency. Figure 6a shows the experimental setup used to detect fluorescent particles on the sensors fabricated for this study. Light from a 633-nm single mode laser was coupled on-chip by aligning the excitation waveguide of sensor with a single mode optical fiber. Fluorescent microbeads (FluoSpheres™ F8806, Invitrogen, Carlsbad, CA, USA), which had a nominal bead diameter of 0.2 µm and were contained within a solution of deionized water at a concentration of 1 × 10^7^ beads/mL, were then introduced into the hollow-core ARROW and pulled through the waveguide with a negative pressure on one end of the hollow waveguide. The microbeads passed through the excitation light and emitted a fluorescent signal that was guided off-chip to an APD, where it was detected.

Figure 6b shows the data that was collected for one of the bARROW sensors tested in this study. Each peak in the graph represents a fluorescence signal from an individual microbead reaching the APD. Similar data was collected for unannealed singe-oxide ARROW sensors, annealed single-oxide ARROW sensors, bARROW sensors with 6-µm tall cores, and bARROW sensors with 7.2-µm tall cores. The noise floor, average peak height, and SNR were calculated for each sensor tested and the average SNR is reported below.

## 3. Results

Before being used for microbead detection, two of the fluorescence sensors, a single-oxide ARROW sensor with a core oxide thickness of 7.2 µm and a bARROW sensor also with a core oxide thickness of 7.2 µm, had their total optical transmissions (their percent optical throughput from one side of the chip to the other) measured in order to assess their waveguiding efficiency. Before annealing, the single-oxide ARROW sensor recorded an optical throughput of 0.02% for 633 nm light. Upon annealing, the optical throughput increased to 9.0%. The bARROW sensor had an optical throughput of 9.1% without any annealing.

Table 2 shows the results for all the sensors tested in this study. The single-oxide ARROW sensor was tested before any annealing was performed. This sensor yielded an average signal per bead of 15 counts/ms with a noise floor of 4 counts/ms, leading to an SNR of 3.75. This is a very low SNR value and makes it difficult to determine true peaks versus fluctuations in noise. After annealing and retesting, the average bead signal increased to 39.7 for the single-oxide ARROW sensor. This confirms the major effect of water absorption on the single-oxide ARROW sensors [15].

There were six bARROW sensors with a core oxide height of 6.0 µm tested, and they yielded an average SNR of 57.0 without any annealing, which is an 43.6% increase over the annealed single-oxide ARROW sensor. The SNR increase may stem from the fact that these sensors never experienced a 300 °C anneal, which typically causes some damage to the waveguides, such as microscopic cracking in the hollow-core ARROW or in the bottom ARROW layers. The additional cracks in these layers are not necessarily visible, but are large enough to be optically significant and create loss-inducing scattering sites in the waveguides. There were seven bARROW sensors with a 7.2 µm thick core oxide that were also tested and yielded an average SNR of 72, an 81.3% increase over the annealed single-oxide ARROW sensor.

## 4. Discussion and Conclusions

This paper reports on optofluidic fluorescence sensors that have been integrated with a buried ARROW (bARROW) design. Burying the ARROWs on the sensor protected them from negative waveguiding effects caused by moisture absorption in the PECVD oxides of the waveguides. Protecting the waveguides from any water absorption eliminated the need for a destructive 300 °C anneal to expel the moisture from the waveguides. The bARROW sensors showed as much of an 81.3% improvement in SNR over the annealed single-oxide ARROW sensors by removing this annealing step from the post-fabrication process. The additional protection of the cladding also increases the long-term stability of the sensor by preventing any gradual degradation of the waveguides over time due to water uptake.

A new PECVD process recipe was developed to lower the intrinsic stress that existed in the oxide films and successfully fabricate bARROW fluorescence sensors without any visible cracking in the waveguides. Intrinsic stress in the PECVD oxides grown in this study tended to become more tensile with increased thickness. The additional thickness in oxide cause by the addition of a new cladding layer led to high intrinsic stress, which initially caused cracking in the hollow channels of the sensors and was only solved once the lower stress PECVD process recipe was developed. The experiments were complemented by simulations performed to determine the coupling efficiency between the integrated solid-core and hollow-core waveguides versus core oxide thickness, which showed that the exact height of the core oxide is quite forgiving and only needs to lie between 5.5 µm and 7.5 µm to keep the coupling efficiency between the solid-core and the 5.5-μm tall liquid core waveguides high. Also, to prevent any negative water absorption effects from occurring in the bARROW fluorescence sensors, the cladding oxide layer must be thick enough that water does not reach deep enough into the waveguide to cause light to scatter out of the core. For the waveguides tested, a 6-µm cladding layer was sufficient.

## Figures and Tables

**Table 1:** Various plasma-enhanced chemical vapor deposition (PECVD) process recipes used for this study. The recipes are listed in order from top to bottom as they are seen in Figure 4a. The light blue recipe was used for all core oxide growths and the yellow recipe was used for all cladding oxide growths.

Process Recipe	SiH_4_ (sccm)	N_2_O (sccm)	CF_4_ (sccm)	Pressure (mtorr)	Temp (°C)	HF (W)	LF (W)	Index	Growth Rate (nm/min)
Orange	164	88	40	1900	250	16	0	1.459	83.91
Gray	164	88	0	1900	250	25	0	1.489	80.14
Light Blue (Core recipe)	164	88	0	1900	250	16	0	1.515	71.08
Yellow (Cladding recipe)	200	500	0	1900	250	40	20	1.452	99.44
Dark Blue	170	710	0	1500	250	40	20	1.45	94.63
Green	82	44	0	660	250	40	0	1.456	52.03

**Table 2:** Average bead signal obtained for each of the fluorescent sensors that were tested.

Sensor Type	Annealed (Yes/No)	Core Oxide Height (µm)	Average SNR
Single-oxide	No	7.2	3.75
Single-oxide	Yes	7.2	39.7
bARROW	No	6.0	57.0 ± 12.0
bARROW	No	7.2	72.0 ± 22.0
